# Quercetin nanoparticles as a therapeutic approach: pharmacological actions and potential applications in therapy

**DOI:** 10.5114/bta.2024.145258

**Published:** 2024-12-19

**Authors:** Reham Farouk El-sayed Baiomy

**Affiliations:** Zoology Department, Faculty of Science, Zagazig University, Sharkia, Egypt

**Keywords:** quercetin nanoparticles, pharmacological effects, nanomedicine, antioxidant, anticancer

## Abstract

The utilization of quercetin nanoparticles as a novel therapeutic strategy has garnered significant attention in recent years. These nanoparticles offer a unique approach to enhancing delivery and effectiveness while overcoming the drawbacks of quercetin. By exploiting the advantages of nanotechnology, such as increased stability and improved bioavailability, quercetin nanoparticles hold significant potential for developing innovative treatments across various medical fields. Quercetin nanoparticles have emerged as an indispensable component in numerous pharmaceutical and medicinal formulations. They are recognized for their anticancer, antitumor, anti-inflammatory, and antidiabetic properties, making them valuable in addressing allergic reactions, metabolic disorders, inflammatory disorders, cardiovascular diseases, and arthritis. From a pharmacological perspective, quercetin nanoparticles have demonstrated beneficial effects against Alzheimer’s disease, primarily through their inhibitory impact on acetylcholinesterase. Furthermore, these nanoparticles have been scientifically documented to possess antioxidant, anticarcinogenic, hepatoprotective, and cytotoxic activities. This comprehensive review aims to explore the pharmacokinetics and biological activities associated with quercetin nanoparticles. It also highlights their potential as therapeutic agents in treating a wide range of diseases, including Alzheimer’s disease, cancer, and neurodegenerative disorders.

## Introduction

**Fig. 1:**
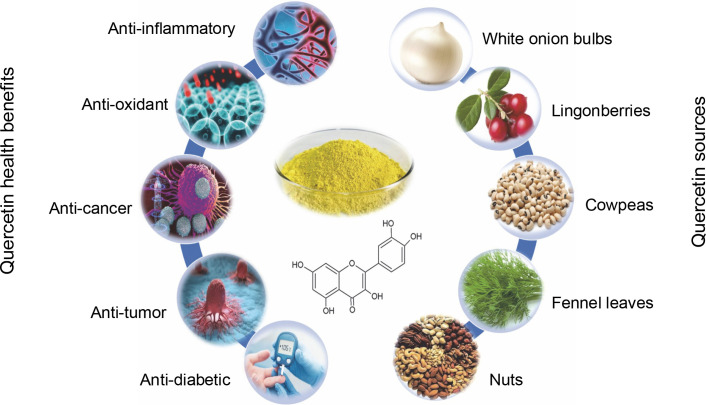
Schematic diagram represents the sources and health benefits of quercetin

Quercetin, a flavonoid, offers a range of health benefits, including anti-inflammatory, antioxidant, and anti-Abd El-cancer properties, as well as cardiovascular and neuroprotective properties (Goyal and Agrawal, 2022; Zhang et al., 2021). The combination of its anti-inflammatory and antioxidant attributes enhances neuronal resilience and reduces cellular mortality in various neurodegenerative ailments, thereby postponing the advancement of diseases (Basile et al., 2022). Notably, quercetin’s ability to inhibit the aggregation of the amyloid-beta peptide Aβ (1–42) – the most harmful form implicated in the amyloid cascade – underscores its significant potential as a neuroprotective agent and antiaging properties (Grewal et al., 2021) – Figure 1.

Additionally, quercetin, despite its numerous therapeutic benefits, is characterized by its insolubility in water and its limited ability to be absorbed through oral administration, leading to a diminished presence in the bloodstream (Mishra and Kulkarni, 2022). Additionally, this polyphenolic compound cannot cross the blood–brain barrier, a critical and highly selective interface between blood capillaries and interstitial fluid, posing a substantial obstacle to central nervous system treatments (Salamone et al., 2021). Its absorption and bioactivity are limited, with bioavailability reported at 17% in rats and a mere 1% in humans (Manzoor et al., 2021). These limitations result in rapid degradation within the bloodstream, necessitating higher doses and prolonged treatment regimens (Michala and Pritsa, 2022). Prolonged use and excessive doses of quercetin, however, may lead to adverse effects (Li et al., 2022).

To address these limitations, numerous synthetic and technological methodologies have been developed, including diverse drug delivery systems (Chen et al., 2023). Among these, nanoparticles have been widely utilized to enhance the transport of quercetin and other natural and synthetic compounds (Lai et al., 2015). Drug nanoparticle technologies have recently gained significant attention as effective methods for improving the solubility of poorly soluble drugs (Müller et al., 2011). Nanoparticles provide several technological advantages as drug carriers, including exceptional stability in biological systems, high drug-carrying capacity, compatibility with both hydrophilic and hydrophobic substances, and the flexibility to support various administration routes, such as oral delivery. These unique properties contribute to improved medication bioavailability and reduced dosing frequency.

Despite advancements in molecular neuroscience, the development of innovative brain-targeted pharmaceuticals has been hindered by the challenge of crossing the blood–brain barrier. However, nanoparticles offer great potential for delivering drugs to the brain and enabling sustained drug release for treating central nervous system disorders (Jiménez-Morales et al., 2022).

Quercetin nanoparticles specifically enhance the solubility and bioavailability of quercetin, making it an effective antioxidant in the cytoplasm, where it protects against reactive oxygen species (ROS) (Zeng et al., 2024). Their unique physicochemical properties allow them to overcome barriers such as the blood–brain barrier. These nanoparticles are widely used in medical research, and their efficacy in combating cancer has been well-documented. They induce apoptosis in cancer cells, further highlighting their therapeutic value (Rahman et al., 2022).

### Quercetin

Quercetin is a primary bioflavonoid that serves as a backbone for many other flavonoids (Terao et al., 1994). The term “quercetin” is derived from the Latin word *quercetum*, meaning “oak forest” (Bhagwat et al., 2014). Quercetin, an organic flavone, is nontoxic in rats at doses of up to 2000 mg/kg body weight (Liu et al., 2020). It is a crystalline, yellow solid with a bitter taste, is insoluble in water, and exists as an aglycone, lacking carbohydrate molecules in its structure. Quercetin also contributes to the vibrant colors of various flowers (Manzoor et al., 2021). This flavonoid is widely found in dietary sources such as onions, apples, tea, and Brassica vegetables, as well as in nuts, seeds, bark, flowers, and leaves. It is also derived from numerous medicinal plants, including *Ginkgo biloba L*., *Petroselinum crispum*, *Polygonum orientale L*., *Nepeta cataria L*., *Mentha canadensis L*., *Crataegus pinnatifida*, *Solanum trilobatum*, and *Hypericum perforatum* (Verma et al., 2013). Additional sources include green vegetables, fruits, and herbs like radish leaves, fennel leaves, pears, cranberries, grapes, tomatoes, kale, capers, and red wine, with capers containing the highest quercetin concentration (234 mg/100 g) (Parhi et al., 2020). Comparative studies have shown that exenatide, at a dosage of 10 μg/kg, demonstrated superior antidiabetic and antihyperlipidemic effects compared to 50 mg/kg doses of both empagliflozin and quercetin. This indicates that quercetin exhibits lower efficacy than exenatide and empagliflozin, largely due to its reduced bioavailability. Transforming quercetin into a nanoparticle form is therefore recommended to substantially enhance its bioavailability and therapeutic effectiveness (Korkmaz and Dik, 2024).

### Drawbacks of quercetin

**Fig. 2:**
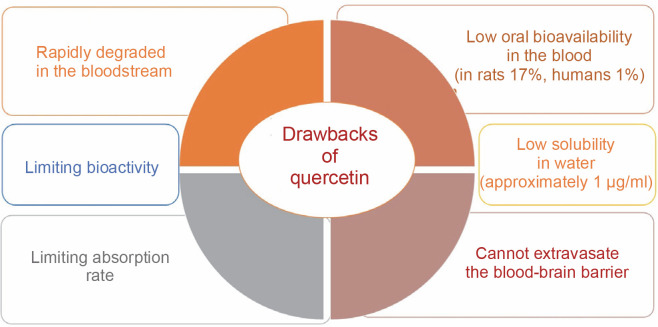
Conceptual illustration shows the drawbacks of quercetin

Quercetin has very low water solubility (approximately 1 μg/ml), which limits its therapeutic potential (Abraham and Acree, 2014) – Figure 2. In gastric and intestinal fluids, its solubility has been reported as 5.5 and 28.9 μg/ml, respectively. To maintain quercetin levels in the blood and tissues for extended periods, its water solubility must be improved, and its metabolism slowed (Nam et al., 2016). According to the biopharmaceutical classification system, quercetin falls into Class IV, characterized by poor water solubility, low permeability, and a short biological half-life (Manzoor et al., 2021). The limited bioavailability of quercetin is due to poor absorption, rapid metabolism, inactivity of its metabolic products, and rapid clearance from the body (Cai et al., 2013). Bioavailability is regarded as the specific amount of a substance that reaches the desired site of action. Furthermore, it is an estimation of the dosage present based on urinary measurements of its constituents and metabolites, whereas, in the case of polyphenol absorption, bioavailability is defined as the amount present in plasma (Almeida et al., 2018).

**Fig. 3:**
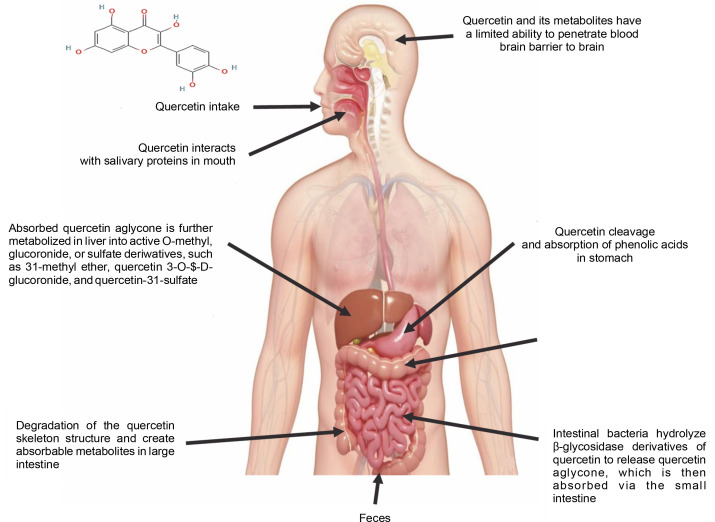
Schematic illustration of how quercetin is metabolized in the body

Quercetin can only be ingested at a rate of roughly 20 mg/day in the human body. However, total plasma concentrations of both free and conjugated quercetin from quercetin-rich foods range from 72 to 193 nmol/l for short-term intake. Long-term dietary use, on the other hand, does not result in plasma accumulation (Wang et al., 2016). Free quercetin was detected in the plasma at a concentration of 0.27 μg/ml following a single intragastric injection of male Sprague-Dawley rats with 50 mg quercetin/kg body weight, and 93% of the quercetin was metabolized within 1 h (Justino et al., 2004). Due to its low solubility, quercetin has an extremely low absorption rate in the gastrointestinal tract, with an oral bioavailability of only 1% in humans (Fujimori et al., 2015). Limited concentrations of free quercetin were found in liver and kidney tissues (less than 8% of total quercetin) following intragastric administration in rats. In the intestine, quercetin can be degraded by intestinal bacteria such as *Eubacterium oxidoreducens*, *Clostridium orbiscindens*, and *Eubacterium ramulus*, with C-ring fission and dehydroxylation resulting in lower molecular weight phenolic compounds that are easily absorbed (Luca et al., 2020) – Figure 3.

### Synthesis of quercetin nanoparticles

**Fig. 4:**
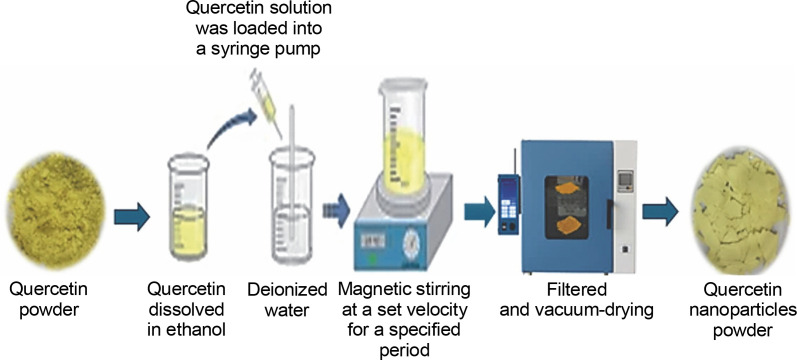
Preparation of quercetin nanoparticles by using nanoprecipitation method

Quercetin nanoparticles are synthesized through a process that reorganizes quercetin molecules into higher-ordered nanoscale structures. This synthesis involves techniques such as precipitation, which enables the formation of stable nanoparticles (Fig. 4). The resulting quercetin nanoparticles exhibit unique properties and enhanced bioavailability, making them promising candidates for applications in medicine, drug delivery, and biotechnology. Advances in nanotechnology and nanomedicine have introduced various methods to improve quercetin's bioavailability, including its conversion into nanoparticles. A high homogenization method and evaporative precipitation into an aqueous solution were employed to create quercetin nano-suspensions, resulting in a higher solubility (Amanzadeh et al., 2019). An antisolvent precipitation technique was used to create quercetin nanoparticles, with stirring speed and flow rate identified as critical factors in particle characteristics (Kakran et al., 2012). Using a nanoprecipitation method, the biocompatibility of quercetin was increased by 20% by adding ethanol to water in a 1 : 3 ratio, yielding nanoparticles 17 nm in size. The solubility of quercetin nanoparticles in water exceeded 55–60 μg/ml after being dissolved for 4–72 h (Manca et al., 2020). Abd El-Rahman and Suhailah (2014) synthesized quercetin nanoparticles using a mixture of ethanol and water at a volume ratio of 1 : 35, employing a fixed flow rate of 8–10 ml/min and magnetic stirring at 1000 rpm. The solution was loaded into a syringe pump for further processing, filtered, and subjected to vacuum drying. The resulting particle diameter was 16.13 nm. Quercetin nanoparticles were also synthesized by Sanad et al. (2024) through a series of steps. Initially, 5 mg of quercetin was dispersed in ethanol within a water bath. This solution was then added to deionized water at a concentration ratio of 1 : 40 and subjected to magnetic agitation at 3000 rpm for 10 min. A fixed flow rate of 10 ml/min was used to introduce the prepared solution into water. The nanoparticles underwent purification and vacuum drying, resulting in particle diameters of 3.63–4.57 nm.

### Characterization of quercetin nanoparticles

**Fig. 5:**
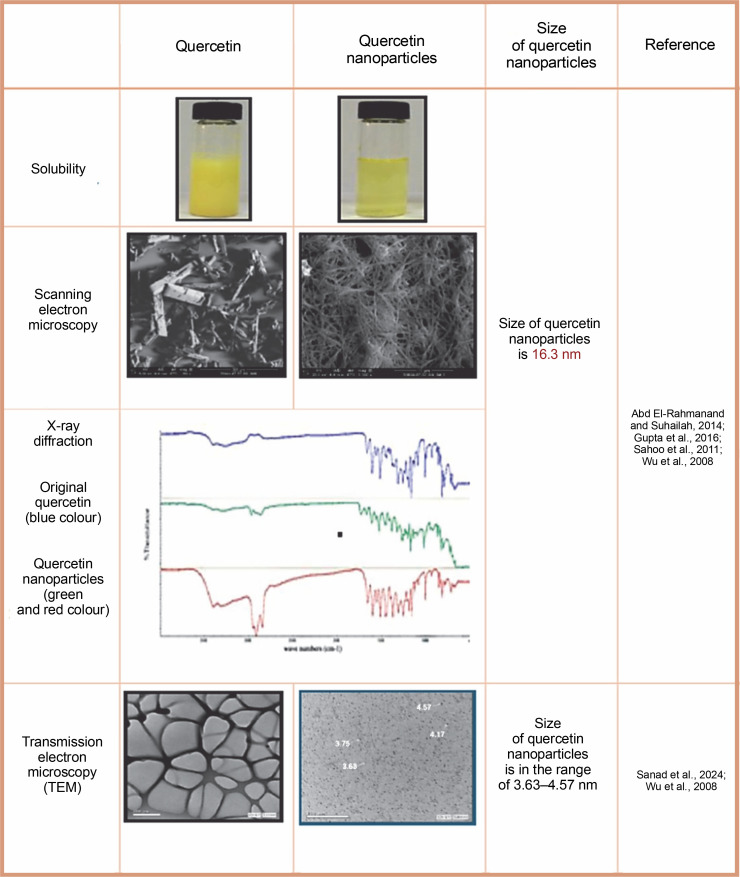
Characterization of quercetin nanoparticles by using X-ray diffraction, solubility and scanning and transmission electron microscope

The characterization of quercetin nanoparticles involves a meticulous evaluation of their properties. This process includes analyzing their physical, chemical, and biological attributes to gain a deeper understanding of their potential (Fig. 5). Key parameters investigated include size, shape, surface charge, stability, drugloading capacity, and release kinetics. Techniques such as dynamic light scattering, transmission electron microscopy, Fourier-transform infrared spectroscopy, and X-ray diffraction are employed for these analyses. Comprehensive characterization helps researchers explore the potential applications of these nanoparticles in drug delivery systems and therapeutic interventions (Abd El-Rahmanand and Suhailah, 2014; Sanad et al., 2024). The manipulation of particle size to decrease it can result in an expansion of the particle surface area, consequently boosting the saturation solubility and dissolution rate of a drug (Muller, 2004). The quercetin nanosuspension displayed a mean size that was within the nanometer range, showcasing its nanoscale properties. When compared to the original quercetin, the quercetin nanocrystals exhibited a notably enhanced dissolution rate. Our investigations have conclusively demonstrated that the increased dissolution rate can be linked to the augmented effective surface area arising from the decreased particle size (Sahoo et al., 2011).

Characterization of both original quercetin powder and synthesized nanoparticles was performed using photon correlation spectroscopy, laser diffraction, scanning electron microscopy, and X-ray diffraction. Results indicated that quercetin nanoparticles averaged 483 nm in size. X-ray diffraction analysis confirmed that lyophilized nanoparticles retained crystalline properties after high-pressure homogenization (Sahoo et al., 2011). The characterization of the novel quercetin nanoparticle systems was conducted through various analytical techniques, including assessments of particle size and morphology, yield, and encapsulation efficiency. Additional methods employed were differential scanning calorimetry, powder X-ray diffraction, Fourier transform infrared spectroscopy, and ^1^H nuclear magnetic resonance. The findings indicated that the particle size of the quercetin nanoparticles was less than 85 nm, with a polydispersity index of less than 0.3, and both yield and encapsulation efficiency exceeded 99% (Wu et al., 2008). The assessment of the morphology and size of the quercetin nanoparticles was performed utilizing transmission electron microscopy and dynamic light scattering methodologies. The findings revealed that the nanoparticles possessed an average diameter within the range of 100–110 nm, along with a low polydispersity index of less than 0.1, suggesting that the quercetin nanoparticles are consistent in their composition (Gupta et al., 2016).

### Quercetin nanoparticles uptake and effects in the brain

Based on the established understanding of nanoparticle science, it is plausible to infer that quercetin nanoparticles could also demonstrate the general be haviors of nanoparticles. Quercetin nanoparticles can enter the brain through two distinct mechanisms: transsynaptic transport following inhalation through the olfactory epithelium and absorption across the blood–brain barrier (Tiwari and Amiji, 2006). Inhalation of nanoparticles through the olfactory section of the nasal cavity allows for their migration to the brain along the pathway of the olfactory bulb (Hopkins et al., 2014). The blood–brain barrier acts as a selectively permeable membrane that delineates the blood from the brain’s interstitial environment, facilitating the regulation of nanoparticle transit by cerebral blood vessels between these two areas (Dotiwala et al., 2018). Quercetin nanoparticle size and shape have also been shown to affect drug delivery into the brain (Blanco et al., 2015). They can travel through the olfactory nerve and bypass the blood–brain barrier to reach the brain. The olfactory nerve connects directly to the brain through long processes. It is potentially possible that inhaled nanoparticles reach the olfactory nerve and travel to the brain through axons (Oberdörster et al., 2004). Quercetin nanoparticle formulations have the potential to penetrate the blood–brain barrier and have neuro-specific effects. They have been shown to have target-specific actions and to be able to pass through the blood-brain barrier (Soni et al., 2016). Quercetin nanoparticle may solubilize lipids in endothelial cell membranes, allowing more efficient transport across the blood–brain barrier. The nanoscale enables access to the cell and its many cellular compartments, including the nucleus (De Jong and Borm, 2008). After crossing the blood-brain barrier, quercetin nanoparticles tend to concentrate in certain brain areas, where they may reach neuronal cells, such as neurons, astrocytes, and microglia (Teleanu et al., 2018). They can distribute medicines throughout the brain spatially and temporally, allowing the treatment of hitherto incurable illnesses. This allows for tissue-specific drug accumulation, unique depot effects, and bypassing of the body's natural barriers, such as the blood-brain barrier (Simkó et al., 2010).

### Pharmaceutical activities of quercetin nanoparticles

#### Antidiabetic activity

**Fig. 6:**
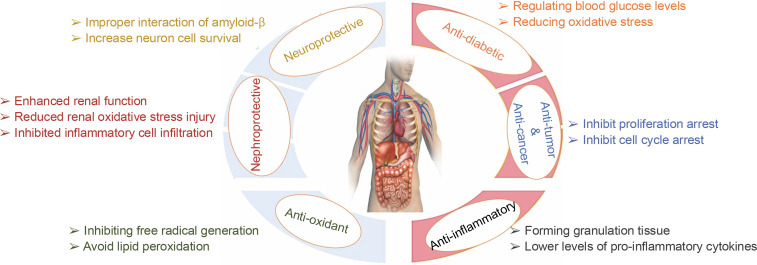
Pharmacological effects of quercetin nanoparticles

Quercetin nanoparticles provide greater protection in alloxan-induced diabetic mice and offer evidence that protection is achieved by regulating blood glucose levels, reducing oxidative stress, and reducing deoxyribonucleic acid damage in tissues – Figure 6. As a result, quercetin nanoparticles may be used as a dietary therapeutic intervention to treat diabetes (Rishitha and Muthuraman, 2018). They were shown to reduce the impact of streptozotocin-induced diabetes and regulate biochemical abnormalities. They have anti-hyperglycemic, homocysteine pathway control, lipid peroxidation reduction, and free radical scavenging properties, and can be utilized to treat diabetic retinopathy (Wang et al., 2020).

#### Antitumor and anticancer activities

The cytotoxic effect of quercetin nanoparticles on human lung adenocarcinoma epithelial cancer cells (A549) demonstrated a substantial reduction in proliferating cell numbers when compared to quercetin (Pimple et al., 2012). After oral therapy, quercetin nanoparticles progressively accumulate drugs in tumor tissues, resulting in tumor cell death. Nanoquercetin has been shown to inhibit proliferation and cell cycle arrest in hepatocarcinoma cells (Bishayee et al., 2015). Nanoquercetin inhibited the progression of breast cancer by inducing apoptosis. Quercetin nanoparticles were used to accomplish the promoted effects on human neuroglioma suppression *via* various cell signaling pathways. Quercetin nanoparticles inhibited neuroglioma development by modifying several signaling pathways, including phosphatidylinositide 3-kinase/protein kinase (PI3K/AKT), extracellular signal-regulated protein kinases 1 and 2 (ERK1/2), and Caspase-3 (Sharma et al., 2015). Quercetin nanoparticles inhibit neuroglioma development through a variety of mechanisms, including autophagy and apoptosis induction via caspase activation as well as AKT (protein kinase B)/mTOR (mammalian target of rapamycin) and antiapoptotic protein (Bcl-2) suppression (Lou et al., 2016).

Quercetin nanoparticles inhibit the development of cervical cancer by inducing apoptosis and autophagy and decreasing cervical cancer cell proliferation by blocking Janus kinase 2 (JAK2) activation. These findings suggested that quercetin nanoparticles could suppress cervical cancer progression by activating Caspase-3 and suppressing Cyclin-D1 and mTOR regulated by Signal Transducer and Activator of Transcription (STAT) 3/5 and PI3K/AKT signaling pathways (Luo et al., 2016). The effects of quercetin nanoparticles on two human breast cancer cell lines (MDA-MB 231 and MCF 7). They found that without causing any toxicity to normal cells, the nanoparticles caused apoptosis and reduced cell proliferation. The authors concluded that the indicated benefits were attributable to quercetin being delivered to the location of action with higher bioavailability (Sarkar et al., 2016). Quercetin nanoparticles are effective in reducing cell numbers of human breast cancer cells (MCF-7 cells) through growth suppression and inducing cell death. Quercetin nanoparticles could induce apoptosis in MCF-7 cells by flow cytometry and gene expression. Flow cytometry results showed that the percentage of necrosis was markedly enhanced in quercetin nanoparticles exposed to MCF-7 cells, which confirms that quercetin nanoparticles can stimulate multiple cell death pathways (Niazvand et al., 2019). In hepatocellular carcinoma *in vivo* and *in vitro*, nano-quercetin showed antitumor properties. The use of quercetin nanoparticles successfully slowed the growth of liver cancer by inhibiting cell proliferation, migration, and colony formation. Apoptosis was also significantly boosted by quercetin nanoparticles. Quercetin nanoparticles increase caspase-9 and caspase-3 cleavage and cause the release of cytochrome c (Cyto-c) in liver cancer cells, leading to apoptosis (Wang et al., 2016). Quercetin nanoparticles also inhibited telomerase reverse transcriptase (hTERT) by lowering the production of activator protein-2 (AP-2 β) and diminishing its interaction with the hTERT promoter. Furthermore, quercetin nanoparticles have an inhibitory role in cyclooxygenase 2 (COX-2) by inhibiting nuclear factor kappa-light-chain-enhancer of activated B cell (NF-κB) nuclear translocation and binding to the COX-2 promoter. By inactivating the caspase/Cyto-c pathway, reducing AP-2 β/hTERT, and blocking the NF-κB/COX-2 signaling pathways, quercetin nanoparticles exhibited an anticancer impact (Ren et al., 2017). Quercetin nanoparticles exhibit antitumor efficacy in the treatment of ovarian, colon, and prostate cancers (Zhao et al., 2017). Quercetin nanoparticles exhibited more efficient anticancer activity than free quercetin in vitro cytotoxicity studies of human lung adenocarcinoma epithelial cancer cells (A549) and a breast cancer cell line (MDA MB 468). In addition, in vivo findings showed that quercetin nanoparticles were more effective than free quercetin in reducing the tumor size in mice bearing lung and breast tumors (Baksi et al., 2018). Forty-eighthour treatment with quercetin nanoparticles significantly decreased malondialdehyde (MDA) levels in C6 glioma cells, which is related to reducing oxidative stress in cells. The findings of this study revealed that quercetin’s cellular uptake and antioxidant activity are improved by small-sized quercetin nanoparticles in C6 glioma cells (Ersoz et al., 2020) – Table 1.

**Table 1:** Action mechanism of quercetin nanoparticles on treating different types of cancer

Cancer type	Mechanism of action	References
Lung adenocarcinoma (A549)	substantial reduction in proliferating cell numbers	Pimple et al., 2012
Neuroglioma	modifying PI3K/AKT, ERK1/2, and Caspase-3; autophagy and apoptosis induction via caspase activation as well as AKT /mTOR and Bcl-2 suppression	Lou et al., 2016
Cervical cancer	inducing apoptosis and autophagy; decreasing cell proliferation by blocking JAK2 activation; activating Caspase-3 and suppressing Cyclin-D1 and mTOR regulated by (STAT) 3/5 and PI3K/AKT signaling pathways	Luo et al., 2016
Breast cancer (MDA-MB 231 and MCF 7)	inducing apoptosis by flow cytometry and gene expression; reduced cell proliferation; growth suppression; stimulate multiple cell death pathways	Sarkar et al., 2016 Sharma et al., 2015
Liver cancer	inhibit proliferation and cell cycle arrest, migration, and colony formation; increase caspase-9 and caspase-3 cleavage; releasing Cyto-c in cells, leading to apoptosis; inhibition of hTERT; lowering the production of AP-2 β and diminishing its interaction with the hTERT promoter; inhibition of COX-2; inhibiting NF-B nuclear translocation and binding to the COX-2 promoter; inactivating the caspase/Cyto-c pathway, reducing AP-2β /hTERT, and blocking the NF-B/COX-2 signaling pathways	Bishayee et al., 2015 Ren et al., 2017 Wang et al., 2016
C6 glioma cells	decreasing MDA levels; reducing oxidative stress	Han et al., 2018

#### Anti-inflammatory activity

**Fig. 7:**
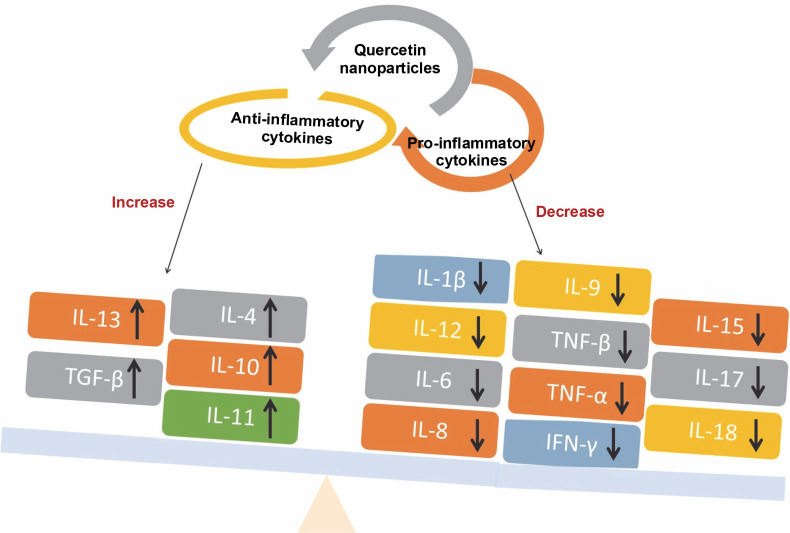
Schematic diagram illustrating the levels of pro-inflammatory and anti-inflammatory cytokines that indicate the anti-inflammatory activity associated with quercetin nanoparticles

Quercetin nanoparticles were able to exert their anti-inflammatory activity locally due to the specific delivery of quercetin nanoparticles to the colon (Castangia et al., 2015). Quercetin nanoparticles had anti-inflammatory effects and estrogenic effects on the ovary (Abd El-Fattah et al., 2017). They found that, because of their better bioavailability, quercetin nanoparticles (10 mg/kg) showed almost identical effects in terms of improvement as free quercetin at a high dose (50 mg/kg). Animals treated with quercetin nanoparticles had significantly lower levels of pro-inflammatory cytokines e.g. IL-1β, IL-12, IL-6, IL-8, and TNF-β than control animals, resulting in less severe endotoxemia symptoms (Penalva et al., 2017) – Figure 7. Quercetin nanoparticles can reduce inflammatory responses and improve wound healing by forming granulation tissue compared with the untreated group. As a result, biogenic nanoparticles are safe, ecofriendly, and simple to synthesize, and they may be regarded as an alternative regimen for inflammatory therapy (Alemzadeh et al., 2022).

**Fig. 8:**
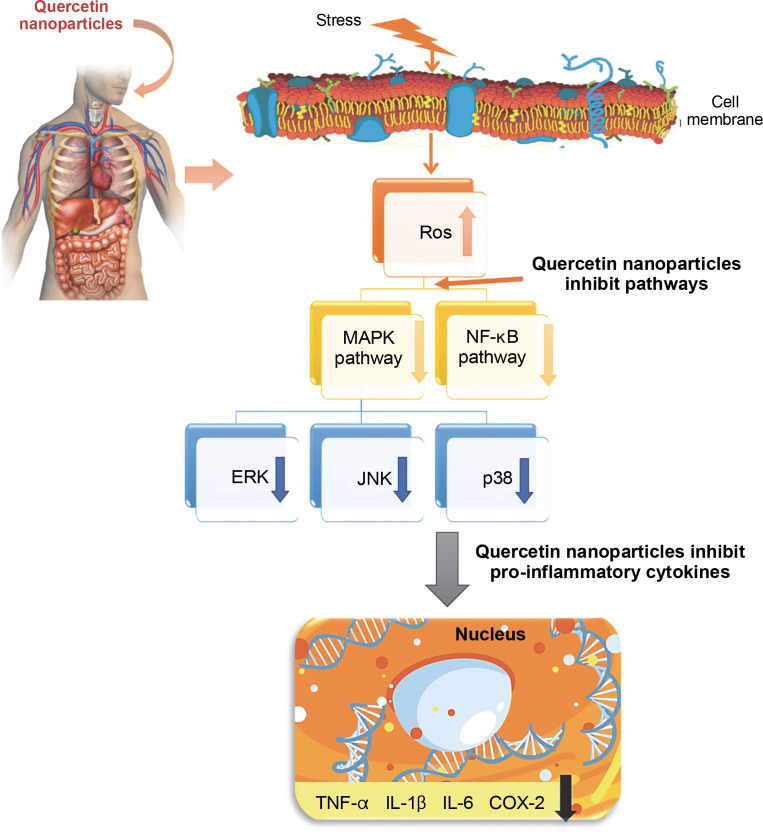
Schematic diagram showing the anti-inflammatory mechanism of quercetin nanoparticles

The anti-inflammatory mechanism of quercetin has been shown to involve the downregulation of pro-inflammatory cytokines such as TNF-α, IL-1β, IL-6, and COX-2 through the inhibition of the MAPK and NF-κB signaling pathways (Liu et al., 2012) – Figure 8. Additionally, quercetin is capable of reducing the formation of reactive oxygen species (ROS). It has been observed that quercetin can diminish the production of inflammatory mediators including IL-1β, IL-6, IL-8, and TNF-α (Xiong et al., 2019). The presence of hydroxy groups in flavonols has been identified as a key factor contributing to their notable anti-inflammatory properties. Furthermore, research indicates that quercetin exerts its anti-inflammatory effects by inhibiting the expression of cytokines and nitric oxide synthase, which is mediated by the suppression of the NF-κB pathway, while not affecting the activity of terminal-c-Jun N kinase. The proinflammatory cytokine TNF-α and the activation of intracellular mediators such as NF-κB p65 in tissues can be effectively inhibited. Moreover, quercetin has been evaluated for its ability to prevent cell apoptosis, demonstrating a capacity to suppress the expression of caspase-3 (Sujana et al., 2021).

#### Antioxidant activity

The antioxidant activity of the quercetin nanoparticles was more efficient than pure quercetin at 2,2-diphenyl-1-picrylhydrazyl (DPPH) scavenging, antisuperoxide formation, superoxide anion scavenging, and anti-lipid peroxidation (Wang et al., 2016). They have the potential to improve cellular function by inhibiting free radical generation or scavenging to avoid lipid per-oxidation and oxidative DNA damage (Bennet and Kim, 2013). The impact of quercetin nanoparticles on increased glutathione peroxidase (GPx) activity may be ascribed to an increase in quercetin bioavailability, whereas catalase (CAT) activity increased considerably, indicating, that most likely, an adaptive response to free radical damage (Suke et al., 2018). They also had strong antioxidant properties and controlled release under a simulated gastrointestinal environment (Barbosa et al., 2019). Quercetin nanoparticles’ excellent biocompatibility and capacity to prevent the oxidative effect of hydrogen peroxide on cells were demonstrated in vitro using keratinocytes, indicating that they might be used to treat skin diseases (Manca et al., 2020). The biological activity of quercetin nanoparticles indicated that they have more antioxidant activity than free quercetin (Moon et al., 2021).

### Clinical trials of therapeutic benefits of quercetin nanoparticles

#### Neuroprotective activity

Quercetin nanoparticles may be a potential component in controlling cerebral ischemia-reperfusion-induced oxidative damage in neuronal cells (Das et al., 2008). They inhibit the degeneration of cholinergic neurons in rats (Phachonpai et al., 2010). Quercetin nanoparticles can reduce neurotoxicity caused by the improper interaction of amyloid-β and increase neuron cell survival. The findings of behavioral testing showed that injecting quercetin nanoparticles into mice improved cognition and memory. The developed quercetin-based nanoscale drug delivery carriers improve the therapeutic index while reducing adverse effects (Sun et al., 2016). Nano-quercetin offered substantial protection against hydrogen peroxide-induced oxidative stress damage in human neuroblastoma cell line (SH-SY5Y) and 6-hydroxydopamine (6-OHDA)-induced neurotoxicity in rat brain synaptosomes (Aluani et al., 2017). The oral administration of quercetin nanoparticles enhanced cognitive and memory deficits. These findings appeared to be linked to a reduction in the expression of the hippocampal astrocyte marker glial fibrillary acidic protein (Bennet and Kim, 2013). Quercetin nanoparticles have antioxidant, anti-inflammatory, and anti-apoptotic effects against H_2_O_2_-induced toxicity in adrenal phaeochromocytoma cells (which showed sympathetic neuron properties), and on the other hand, quercetin nanoparticles have magnetic properties and can be directed to a specific tissue, so we propose quercetin nanoparticles as a suitable candidate for neural treatment (Yarjanli et al., 2019). The capacity of quercetin nanoparticles to induce neuroprotective effects was investigated utilizing Aβ (1–42) peptide as an in vitro Alzheimer’s disease model. The ability of quercetin nanoparticles to prevent fibril production has also been established. Because of their better capacity for site-specific delivery of quercetin into the brain and their improved inhibitory impact on amyloid-beta aggregation, quercetin nanoparticles are promising for neurological illness treatment, such as Alzheimer’s disease (Pinheiro et al., 2020; Sanad et al., 2023). The administration of quercetin nanoparticles protects brain cells from arsenic-induced damage. The mechanism of the quercetin nanoparticles transport across the blood–brain barrier appears to involve endocytotic absorption by brain capillary endothelial cells, followed either by quercetin release in these cells and diffusion into the brain or by transcytosis (Ghosh et al., 2009). They also protect neurons from amyloid-beta fibrillation in a thioflavin T binding experiment (Pinheiro et al., 2020). Quercetin nanoparticles provide a preventative approach against the development of Alzheimer’s disease (Fig. 8). Quercetin nanoparticles outperformed the quercetin-treated group of rats in terms of efficacy, suggesting that the improved efficacy is attributable to a longer residence period in systemic circulation and greater bioavailability. Quercetin nanoparticles significantly reduced MDA and acetylcholine esterase (AChE) levels while increasing brain CAT and glutathione (GSH) (Palle and Neerati, 2017). Quercetin nanoparticles improved brain oxidation by increasing antioxidant enzyme activity and reducing pro-oxidant effects. Quercetin nanoparticles reduce ROS, protein carbonyl, and myeloperoxidase (MPO). In addition, they increased the activity of GPx and AChE (Eman et al., 2018). Treatment with quercetin nanoparticles improved the gammaaminobutyric acid (GABA) level in the cerebellum. The quercetin nanoparticles increased superoxide dismutase activity, and GSH levels, and reduced lipid peroxidation in the hippocampus region. This finding revealed that enhanced antioxidant enzyme activities can lead to decreased intracellular H_2_O_2_ generation, which, when combined with an increase in GSH levels, can reduce lipid peroxidation (Ghaffari et al., 2018; Sanad et al., 2024). In rats with aluminum-induced dementia, quercetin nanoparticles showed a significant increase in memory retention. Furthermore, the persistence of lipid peroxidation, GSH, and nitrite levels in the brain homogenates of these rats confirmed quercetin nanoparticles’ effective targeting of the central nervous system (Dhawan et al., 2011). Nanoquercetin therapy demonstrated protective benefits by reducing ROS production in both young and old rat brain areas. As a result, the lower ROS must be able to compensate for the higher conjugated diene production and low GSH and antioxidant enzyme levels (Ghosh et al., 2013). Quercetin nanoparticles were demonstrated to have a cognitively attenuating impact on pentylenetetrazole-induced neurocognitive deficits, as well as to improve biochemical changes. Quercetin nanoparticles also show that they have direct and indirect endogenous antioxidant (i.e., increase reduced GSH) action, anti-inflammatory (i.e., decrease lipid peroxidation), and neurotransmitter regulating (i.e., decrease AChE activity) effects. Because of its potential antioxidative, antilipid peroxidative, and acetylcholinesterase inhibitory effects, quercetin nanoparticles might be utilized as future nanomedicine for different neurodegenerative diseases (Rishitha and Muthuraman, 2018). Quercetin nanoparticles enhance memory retention in rats with aluminum-induced dementia, implying that quercetin nanoparticles might be useful for brain targets and Alzheimer’s disease (Dhawan et al., 2011). They have the potential to be employed as an autophagy inducers in the treatment of Alzheimer’s disease. They can more effectively activate autophagy in SHSY-5Y cells (a human-derived cell line used as models of neuronal function and differentiation), increase autophagosomelysosome fusion, speed up the clearance of intracellular amyloid-β (Aβ), and protect SH-SY5Y cells against Aβ-induced cytotoxicity. They work in concert to increase SHSY-5Y autophagy, remove Aβ, and reduce Aβ-mediated cytotoxicity (Sudheesh and Pawar, 2022). They were used to regulate Aβ42 assembly and showed multifunctional effects, including inhibition of Aβ aggregation, destabilization of Aβ fibrils, reduction of Ab-induced oxidative stress, and reduction of Aβ-mediated cytotoxicity. As a result, quercetin nanoparticles with increased bioavailability might serve as a multifunctional therapeutic agent for amyloid-related illnesses (Han et al., 2018). Quercetin nanoparticles that target the blood–brain barrier while also protecting neurons from amyloid-beta fibrillation in a thioflavin T binding experiment showed to be an effective method of quercetin administration and a potential technique for future Alzheimer’s disease treatments (Martano et al., 2022). The use of a nanoparticle form of quercetin with a higher bioavailability could account for the significant effect on astrogliosis. Astrocytes are thought to be involved in Aβ clearance and they may also produce interleukin-33, which promotes the engulfment of microglial synapses and the development of neural circuits (Ghaffari et al., 2018). Astrogliosis develops in response to brain damage and Aβ accumulation, is triggered by inflammatory mediators like nitric oxide and cytokines, and has been linked to the neurodegenerative disease Alzheimer’s disease (Selvakumar et al., 2018). Astrogliosis may be a protective mechanism in Alzheimer’s disease, as astrocytes can remove Aβ and reduce the formation of amyloid plaques (Kaur et al., 2019). The quercetin nanoparticles treated group of rats showed the predominance of normal microglia with only a few dark microglia. This could be explained by quercetin nanoparticles’ ability to prevent oxidative stress, neuroinflammation, and the formation of amyloid plaques. As a result, modulating phagocytic processes has opened previously unknown avenues for the development of novel therapeutics that promote CNS repair and regeneration (Hayden et al., 2018). Quercetin nanoparticles significantly protected and alleviated cerebellar tissues affected by tartrazine-induced necrosis and pyknosis of Purkinje cells. The protective effects of quercetin nanoparticles against tartrazine-induced neuro-toxicities may be due to the antioxidant, anti-inflammatory, and antiapoptotic effects (Niazvand et al., 2019). Nanocapsulated quercetin exhibited neuroprotective properties and protected brain mitochondria from the pathological state of cerebral ischemia-reperfusion (Ghosh et al., 2013).

#### Hepatoprotective activity

Quercetin nanoparticles prevented arsenite-induced declines in antioxidant levels in the liver. Arsenic caused a significant reduction in the microviscosity of liver cell membranes, and treatment with quercetin nanoparticles provided unique protection against this loss. There is a significant link between mitochondrial arsenic and conjugated diene concentrations in liver cells (Ghosh et al., 2009). Quercetin nanoparticles caused fast regeneration of hepatic biomarkers, as well as down-regulation of serum enzyme parameters and a substantial increase in hepatocytes even when administered after toxin-induced hepatic damage. According to these findings, they substantially increased solubility, which improved the bioavailability and survival of damaged hepatic cells (Chen et al., 2023). Nanoquercetin was more successful in restoring liver membrane integrity when compared to naked quercetin, as evidenced by substantially lower serum markers such as alanine transaminase, aspartate aminotransferase, alkaline phosphatase, and lactate dehydrogenase. The findings of decreased collagen levels and histopathology also demonstrated that the benefits of nanoquercetin are far superior to those of naked quercetin. Biochemical factors such as antioxidant defense enzymes also support this assertion. Consequently, the findings clearly show that nanoquercetin not only provides considerable hepatoprotection against liver cirrhosis but also lowers the necessary concentration (1,000 to 10,000-fold lower) by improving bioavailability (Verma et al., 2016). The administration of quercetin nanoparticles more efficiently decreased the rate of ROS formation and lipid peroxidation, improved cell viability, mitochondrial membrane potential, and GSH level, and demonstrated a significant hepatoprotective effect by lowering levels of aspartate aminotransferase, alanine aminotransferase, and alkaline phosphatase. It is claimed that quercetin nanoparticles are a viable choice for medication administration because they improve the hepatoprotective action of quercetin against the cytotoxic effects of aflatoxin (Eftekhari et al., 2018). Nanoquercetin therapy showed significant antihepatotoxic efficacy against chlorpyrifos-induced hepatic damage and immunotoxicity by improving all liver enzymes, antioxidant enzymes, and immune response parameters, as verified by histological analysis (Suke et al., 2018). As a result, nano-quercetin may provide a cushion for a longer treatment alternative against toxin-induced hepatotoxicity while avoiding severe side effects. They with excellent biodegradability and biocompatibility can enhance the bioavailability of poorly soluble quercetin and significantly inhibit hepatic fibrosis. They may be taken up by hepatic stellate cells via their binding affinity with integrin alpha V and integrin beta 3 (αvβ3), specifically accumulate in the fibrotic liver in carbon tetrachlorideinduced mouse models and significantly suppress the activation of hepatic stellate cells both *in vivo* and *in vitro* (Zhang et al., 2019). Nanoquercetin has been shown to protect rats against liver injury and damage. In the rat liver, it has high bioactivity and bioavailability, and it significantly reduces the liver index and pathological alterations. The serum levels of glutamic-pyruvic transaminase, glutamic-oxaloacetic transaminase, and direct bilirubin were substantially higher following nanoquercetin administration than following pure quercetin treatment (Liu et al., 2020).

#### Nephroprotective activity

Quercetin nanoparticles significantly ameliorated pathological damage to the kidney, enhanced renal function, reduced renal oxidative stress injury, inhibited inflammatory cell infiltration and downregulated the expression of intercellular adhesion molecular-1 (ICAM-1) (Tong et al., 2022). The results of the morphological and serum biochemical tests showed that giving quercetin nanoparticles to diabetic rats has a positive effect on cell regeneration in the injured kidney. As a result, it may be inferred that quercetin nanoparticles have a preventative and curative impact on streptozotocin-induced diabetes in rats and that they can be utilized as a natural herbal medication to preserve kidney and pancreas cells (Al-Jameel and Abd El-Rahman, 2017).

#### Cardioprotective activity

Quercetin nanoparticles restored normal cardiac contractility, demonstrating cardioprotective effectiveness in cardiac ischemia and a disintegrating impact on peroxynitrite molecules (Soloviev et al., 2002). Because of its enhanced oxidative stress suppression, the administration of quercetin nanoparticles resulted in the maintenance of mitochondrial function and Adenosine triphosphate production. These findings suggest that this approach has potential for the treatment of oxidative stress-related cardiac diseases (Lozano et al., 2019).

#### Stomach protective activity

Nanoquercetin was shown to be 20 times more effective than quercetin in preventing ethanol-induced gastric ulcers. The administration of nano-quercetin significantly blocked the synthesis and release of matrix metalloproteinase (MMP)-9, as well as the infiltration of inflammatory cells and oxidative damage in rat gastric tissues. Nanoquercetin, ascompared to quercetin, preserved mitochondrial integrity and size, as well as mitochondrial activities, in rat gastric tissues via regulating succinate dehydrogenase. Furthermore, both quercetin and nanoquercetin inhibited poly ADP-ribose polymerase 1 (PARP-1) and apoptosis during ethanol-induced gastric ulcer prevention. Nanoquercetin had a stronger effect than quercetin on the expression of enzymes, including cyclooxygenase and nitric oxide synthase (Chakraborty et al., 2012).

## Conclusion

The use of quercetin nanoparticles as a novel therapeutic strategy shows immense potential for treating various diseases. These nanoparticles offer advantages such as enhanced solubility, prolonged circulation time, and improved cellular uptake. The ability to modify their surface properties allows targeted drug delivery, minimizing off-target effects and maximizing therapeutic outcomes. With ongoing research, quercetin nanoparticles are poised to revolutionize medicine and provide effective treatment options for numerous ailments.

## Data Availability

Not applicable.
